# Chronic Lower Extremity Ischemia and Its Association with the Frailty Syndrome in Patients with Diabetes

**DOI:** 10.3390/ijerph17249339

**Published:** 2020-12-14

**Authors:** Grzegorz K. Jakubiak, Natalia Pawlas, Grzegorz Cieślar, Agata Stanek

**Affiliations:** 1 Department and Clinic of Internal Medicine, Angiology and Physical Medicine, Specialistic Hospital No. 2 in Bytom, 41-902 Bytom, Poland; grzegorz.k.jakubiak@gmail.com; 2 Department of Pharmacology, Faculty of Medical Sciences in Zabrze, Medical University of Silesia, 41-800 Zabrze, Poland; natalia.pawlas@sum.edu.pl; 3 Department and Clinic of Internal Medicine, Angiology and Physical Medicine, Faculty of Medical Sciences in Zabrze, Medical University of Silesia, 41-902 Bytom, Poland; cieslar1@tlen.pl

**Keywords:** diabetes, Frailty, lower limb ischemia, peripheral arterial disease

## Abstract

Diabetes mellitus is an important risk factor for the development of cardiovascular diseases. Peripheral arterial disease affecting lower limb arteries is one of the clinical manifestations of atherosclerosis. The frailty syndrome (Frailty) is a problem associated with diminution of physiological reserves. The ankle-brachial index is a commonly used tool for diagnosing peripheral arterial disease (PAD). The usefulness of the ankle-brachial index (ABI) is limited in people with diabetes because of calcification of the middle layer of arteries. In this population, toe-brachial index should be measured. Frailty may be associated with worse prognosis for patients undergoing revascularization. Amputation may be an important factor leading to the development of Frailty. The risk of amputation and the prognosis after revascularization may be modified by some medications and blood glucose levels. The purpose of this paper is to review the literature about the association between PAD, especially in patients living with diabetes and Frailty.

## 1. Introduction

The increasing prevalence of type 2 diabetes mellitus is one of the most important problems for public health worldwide, especially in developed countries. According to data collected and published by Khan et al., 462 million individuals have been affected by type 2 diabetes mellitus in 2017 (6.28% of the world’s population) [1]. Diabetes mellitus is a significant risk factor for the development of atherosclerosis [2], near such risk factors as dyslipidemia [3], arterial hypertension [4], poor physical activity [5], tobacco smoking [6] and obesity [7]. Diabetes also significantly increases the risk of cardiovascular events. In a seven-year follow-up study, the risk of a first heart attack was found to be 20.2% for people with diabetes and 3.5% for people without diabetes [8].

As a result of atherosclerosis, to which diabetes predisposes, ischemic heart disease, stenosis of the carotid and vertebral arteries, chronic ischemia of the lower limbs, chronic intestinal ischemia, or narrowing of the renal arteries may develop, among others [9]. Chronic lower extremity ischemia is a manifestation of peripheral arterial disease (PAD) affecting arteries responsible for blood supply of lower limb. It may result from stenosis (narrowing) or occlusion (complete blockage) of vascular lumen [10]. Atherosclerosis is responsible for approximately 95% of cases and other factors such as vasculitis, as well as genetic and traumatic causes for approximately 5% [11].

The prevalence of lifestyle diseases in the population, including the clinically evident consequences of atherosclerosis, increases with age. In view of the progressive aging of the population in highly developed countries, the importance of geriatric problems, such as the frailty syndrome (Frailty), for healthcare systems is increasing [12]. Frailty is a geriatric syndrome that results from a multi-system reduction in reserves, deterioration of the ability to adapt to stressful situations, and thus an increased risk of such adverse phenomena as infections, falls, deterioration of cognitive abilities, dependence on other people or institutions [13,14].

The purpose of this paper is to present a review of the literature on the current state of knowledge about chronic lower limb ischemia and its association with diabetes mellitus as an important factor which can lead to worsening of quality of life and Frailty. We also described how Frailty in patients with chronic lower limb ischemia affects the prognosis and the effectiveness of treatment.

## 2. Frailty

Fried et al. proposed that at least three of the following five conditions should be met as a criterion for the diagnosis of Frailty: (1) weight loss, unintentional, of ≥10 pounds in prior year or, at follow-up, of ≥5% of body weight in prior year (by direct measurement of weight); (2) grip strength in the lowest 20% at baseline, adjusted for gender and body mass index; (3) low energy; (4) slowed walking speed; and (5) low physical activity [13]. It should be emphasized that the concept of Frailty is clearly separated from the terms of comorbidity and disability, although these conditions may coexist [15].

The pathogenesis of Frailty is multifactorial. Such mechanisms as decline in metabolism, inflammation, DNA damage, hormone dysregulation, epigenetic alterations, loss of proteostasis, and senescence and stem cell exhaustion as well as environmental factors are involved in development of Frailty [16]. The contribution of polypharmacy, malnutrition, inactivity and social isolation in the development of Frailty also has been noted [17].

The overall prevalence of Frailty in Europe is assessed as 7.7% in the population aged above 50 years (from 3.0% in Switzerland to 15.6% in Portugal) [18]. It has been documented that in European population women are slightly more predisposed to Frailty (OR 1.56, 95% CI: 1.51–1.62) and comorbidity (OR 1.11, 95% CI: 1.07–1.15), and sex difference increases with age [19].

In the population-based study conducted by Chao et al. involving 525,368 patients with diabetes, 64.4% were not frail, while 28.5%, 6.6%, and 0.6% had one, two, and three or more features of Frailty, respectively. It has also been observed that factors such as age, feminine gender, smoking and alcoholism were significantly associated with increased susceptibility to the development of Frailty [20].

The presence of Frailty increases the risk of more severe course of certain diseases or predisposes to the development of additional health problems. For example, it has been proven that it increases risk of progression of diabetic kidney disease to end-stage renal disease [21]. Patients with diabetes and the features of Frailty are more likely to develop urolithiasis [20]. According to the results of the meta-analysis conducted by Ida et al. in patients living with diabetes there is a relationship between the incidence of Frailty and the mortality and risk of hospitalization [22].

One of the commonly used tools for assessing the severity of Frailty, including in the field of research on the population of patients undergoing endovascular treatment or open vascular surgery procedures, is the 11-factor modified Frailty Index (mFI) [23,24,25]. Eleven elements are taken into account when calculating the index. The presence of each of these factors is assigned one point, and if there are none, zero points. Then the sum of the points should be divided by eleven, obtaining the mFI value in the range of 0.0–1.0. Factors taken into account include: non-independent functional status, history of diabetes mellitus, history of chronic obstructive pulmonary disease, history of congestive heart failure, history of myocardial infarction, history of percutaneous coronary intervention, cardiac surgery, or angina, hypertension requiring the use of medication, peripheral vascular disease or rest pain, impaired sensorium, transient ischemic attack or cerebrovascular accident without residual deficit, cerebrovascular accident with deficit [26]. The exact interpretation of the mFI value is ambiguous. According to Pandit et al., Frailty status is defined with a cut off limit of 0.27 [27]. Gonzalez et al. used three categories with the following thresholds: robust 0.12, prefrail 0.25, and frail 0.4 [28].

Presence of the features of Frailty in patients living with diabetes should be taken into consideration in the therapeutic process. Frailty better than age reflects functional and health status of elderly people [29]. The presence of Frailty adversely affects the course of diabetes. Both hyperglycemia and episodes of hypoglycemia are associated with acceleration of changes in the brain, which contribute to further deterioration of the patient’s functional and cognitive state [30,31]. It has been documented that both hypoglycemia and hyperglycemia are associated with increased risk of fracture in Japanese patients living with type 2 diabetes mellitus [32].

Maddaloni et al. presented an interesting commentary elucidating the crucial role of taking into consideration the presence of features of Frailty in patients living with type 2 diabetes in therapeutic strategy. They noted that clinical trials for frail patients living with diabetes and the preparation of a specific scale for the assessment of Frailty in patients living with diabetes are needed [33].

## 3. Pathology and Epidemiology of PAD

Atherosclerosis is a chronic inflammatory disease, and processes such as endothelial dysfunction, oxidative stress, oxidative modification of low-density lipoproteins, intimal lipid deposition, smooth muscle cells proliferation, foam cells formation, apoptosis and necrosis, and local and systemic inflammatory response are involved in pathogenesis of atherosclerosis [34]. Atherosclerosis is a pathological process involving the wall of arteries, which leads to plaque formation, narrowing of lumen of the vessel and chronic ischemia of the supplied organ. It may result in clinical manifestation of such diseases as coronary heart disease, stroke and peripheral arterial disease (PAD) [35]. It is worth noting that the lack of mobility, which is an important element in the development of Frailty, is associated with an additional contribution to the reduction of blood flow dynamics in the lower extremities. It is noteworthy that diabetes is not only a risk factor for occurrence atherosclerosis but also for acceleration its development [36]. Diabetes affects the function of endothelial cells, smooth muscle, and platelets, contributing to plaque instability, rupture, and cardiovascular events [37].

Hyperglycemia and insulin resistance are two significant factors responsible for promoting of cardiovascular diseases development in patients with diabetes mellitus through such biochemical mechanisms as increased aldose reductase substrate conversion, formation of advanced glycation end-products, activation of protein kinase C, protein modification by N-acetylglucosamine, activation of the transcription factor NFAT, activation of NLRP3 inflammasome and peptidylarginine deaminase 4, and NETosis activation [38]. NETosis is a form of neutrophil cells death which participates in immune response against pathogens. During NETosis, neutrophils release neutrophil extracellular traps (NETs), which can capture and kill pathogens [39]. Reactive oxygen species and cellular death are associated with the majority of biochemical alterations promoting development of cardiovascular disease in the course of diabetes [40].

A typical symptom for chronic lower extremity ischemia is intermittent claudication, which is characterized by limb (especially calf) pain associated with walking and relieved by rest [41]. There are two scales widely used in clinical practice to describe the severity of claudication: the Fontaine classification and the Rutherford classification, presented in Table 1 [42,43,44]. Scales such as, among others, Bollinger’s angiographic classification, Graziani’s morphologic categorization and Society for Vascular Surgery WIfI (wound, ischemia, foot infection) classification are also used to assess the severity of the disease [45]. The most severe form of peripheral arterial disease affecting lower limb is critical limb ischemia (CLI) characterized by rest pain, ulcer and tissue necrosis. It may be complicated by infection. In some cases, amputation may be necessary [46].

The prevalence of PAD on the European continent has been estimated at 5.3% (40 million subjects) [47], while that in the European Union (EU) has been estimated at 3.4% (17 million patients) [48]. It is estimated that prevalence of PAD among non-EU citizens on the European continent is 9.2% [49]. Song et al. performed meta-analysis of 118 papers. It has been assessed that 236.62 million people globally aged 25 years and older were living with peripheral arterial disease in 2015, meaning that the global prevalence of peripheral arterial disease in this population is 5.56% [50]. The PANDORA study, in which 10,287 people from six European countries were enrolled, showed significant differences in PAD prevalence between individual countries: Greece (28.0%), Italy (22.9%), France (12.2%), Switzerland (12.2%), The Netherlands (8.1%) and Belgium (7.0%) [51].

Olinic et al. distinguished the following risk factors for the progression of PAD: tobacco use, physical inactivity, unhealthy diet, harmful use of alcohol, hypertension, diabetes, dyslipidemia, obesity, poverty, low educational status, advancing age, gender, genetic disposition, stress, depression, inflammation, lower glomerular filtration rate, higher interleukin-6 levels, presence of coronary artery calcification, long-term air pollution, infection disease [49]. Fowkes et al. performed a systematic review and analysis to assess risk factors for peripheral arterial disease. According to the results of this study the most strongly correlating with PAD risk factors in high-income countries were smoking (current smoker) (OR 2.72, 95% CI: 2.39–3.09), history of other cardiovascular disease such as coronary heart disease or stroke (OR 2.55, 95% CI: 2.16–3.02), smoking in the past (OR 2.03, 95% CI: 1.71–2.41) and diabetes (OR 1.88, 95% CI: 1.66–2.14) [47].

It should be emphasized that the clinical course of peripheral arterial disease in patients with diabetes and those without diabetes shows some differences. In patients living with diabetes, arteries below the knee (such as popliteal, anterior tibial, peroneal and posterior tibial) are more commonly involved [52]. Patients with diabetes show symmetrical, multi-segmental stenosis [53]. These facts are reflected in the difference in the effectiveness of reperfusion therapy between people with and without diabetes. It has been shown that patients living with diabetes present a higher rate of binary restenosis (54.4% vs. 31.5%) and amputation (24.4% vs. 1.5%) at 2 years following peripheral transluminal angioplasty [54]. DeRubertis et al. performed a retrospective analysis of medical history of 291 patients suffering from lower limb ischemia treated by percutaneous intervention. In this study, it is documented that diabetes is associated with reduced effectiveness of primary intervention (patency after 6, 12 and 18 months in patients with diabetes vs. patients without diabetes 82 ± 2%, 53 ± 4%, and 49 ± 4% vs. 88 ± 2%, 71 ± 4%, and 58 ± 4% respectively) with no significant difference in the effectiveness of secondary intervention [55].

The occurrence of suboptimal blood glucose levels is associated with a greater likelihood of developing cardiovascular complications, including peripheral arterial disease. Zhang et al. conducted a meta-analysis of 26 prospective cohort studies. According to the results of this study, the relative risk for the emergence of peripheral arterial disease associated with a 1% increase in glycated hemoglobin is 1.29 (95% CI: 1.18–1.40) [56].

A meta-analysis published in 2017 including 21 studies confirmed that diabetes is a strong factor increasing mortality among patients with PAD (OR 1.89, 95% CI: 1.51–2.35). Taking into consideration only patients with critical limb ischemia, the influence of diabetes on mortality was even stronger (OR 2.38, 95% CI: 1.22–4.63) [57].

## 4. The Significance of Ankle-Brachial Index (ABI) and Toe-Brachial Index (TBI)

The ankle-brachial index (ABI) plays the crucial role in diagnosis of PAD affecting lower limb. It is defined as the ratio of the systolic blood pressure measured at the ankle to that measured at the brachial artery [58]. This term was introduced by Winsor in 1950 [59]. It has been documented that a value of ABI below 0.9 correlates with significant disorder of blood supply of lower extremity, and this makes it possible to diagnose PAD affecting lower limbs [60,61]. According to guidelines prepared by European Society for Vascular Medicine published in 2019, in the absence of mediasclerosis, values of ABI between 0.75 and 0.9 are associated with a mild degree, between 0.5 and 0.75 with a moderate degree, and below 0.5 with a severe degree of PAD affecting lower limbs [11].

Currently, the ABI is important not only in diagnosis of PAD, but also as a valuable indicator of total cardiovascular risk, similar to such parameters as intima-media thickness (IMT) or coronary artery calcium score [62,63]. In a prospective observational study performed on a group of middle-aged Americans by Gupta et al., it was confirmed that low value of ABI is an independent risk factor for heart failure occurrence. ABI not exceeding 0.90 was associated with a 40% increased risk, whereas ABI between 0.91 and 1.00 resulted in a 36% increased risk of heart failure, independently of traditional risk factors [64]. Murabito et al. observed, in a study performed on a group of 674 elderly people, no significant correlation between ABI below 0.9 and increased risk of coronary heart disease and death, while there was a significant relationship between ABI less than 0.9 and increased risk for stroke or transient ischemic attack (HR: 2.0, 95% CI: 1.1–3.7). The authors emphasized a need for confirmation of these results on a larger sample [65].

There are some specific aspects in the interpretation of ABI values in patients with diabetes mellitus that make it less useful in the diagnosis of chronic lower limb ischemia in this population. It is well established that diabetes is strongly associated with a greater predisposition to calcification of the middle layer of the artery causing stiffness of the arterial wall. Calcification of the middle layer of arteries occurs with a frequency of 4.4–15.6% in healthy subjects and 15.7–41.5% in patients with diabetes [66]. Arterial stiffness, as well as neuropathy and foot wounds, is associated with decreased sensitivity of ABI measurement in diagnosis of PAD in patients living with diabetes [67,68]. It is noteworthy that, according to results from the Strong Heart Study published in 2004, ABI exceeding 1.4 is associated with increased mortality similar to ABI below 0.9, due to all-case as well as cardiovascular disease [69]. Thus, the linear relationship between the ABI value and cardiovascular risk in the diabetic population becomes U-shaped.

Singh et al. conducted an interesting analysis of data from 3571 participants of the National Health and Nutritional Examination Survey (NHANES) to assess the relationship between the frequency of the frailty syndrome and the value of the ABI index. The results of this analysis are presented in Table 2 [70]. Alonso-Bouzón et al., analyzing data from the Toledo Study for Healthy Aging, indicated that the ABI value was distributed in the subgroup of participants with Frailty, without Frailty and in the intermediate state called pre-Frailty (persons who met one or two from five criterions of Frailty). The results are shown in Table 3. Of the 1287 participants in the study, 240 people had diabetes. Although the percentage of diabetic patients was greater among frail persons (23.1%) than among both pre-frail (21.0%) and no frail (16.4%), the difference was not statistically significant (*p* = 0.069) [71].

Interesting results were presented by Xue et al. It was shown that the value of the ABI waas significantly lower among the frail group than in the pre-frail group and non-frail group. The outcomes of the study strongly suggested that atherosclerosis was associated with significantly worse walking speed test results achieved by patients. This shows that these patients are more predisposed to poorer mobility, strength and balance, which are features directly associated with criteria for the diagnosis of the frailty syndrome [72]. Similar conclusions were drawn from the results of the ARIC Study, which assessed the relationship of multiple parameters assessing cardiovascular function with a predisposition to Frailty, including echocardiography, ABI, pulse-wave-velocity, spirometry, estimated glomerular filtration rate, hemoglobin concentration, body mass index and bioimpedance [73].

Although it is recommended to use the toe-brachial index instead of the ankle-brachial index in the diagnosis of peripheral arterial disease in the population of patients living with diabetes [11], there are also reports in the literature suggesting that ABI should not be omitted as the screening test in patients with diabetes and with foot ulcer [74]. The toe-brachial index (TBI) is defined as the quotient of systolic blood pressure measured at the big toe versus systolic blood pressure measured at the arm [75]. TBI value below 0.75 is considered a criterion for the diagnosis of PAD according to the International Working Group on the Diabetic Foot (IWGDF) [76]. The European Society of Vascular Medicine defines TBI values below 0.7 as pathological [11]. The measurement of TBI also has a prognostic value. In the prospective observation study involving 741 middle-aged patients with diabetes, it was proven that low TBI value is associated with increased risk for major adverse cardiovascular events and all-cause mortality [77]. There are no results in the literature that clearly link the issue of the frailty syndrome with the value of the toe-brachial index. Investigating this relationship, especially in the population with diabetes, may be an interesting topic for future research.

## 5. Revascularization Treatment

In the treatment of chronic lower limb ischemia, an important role is played by invasive reperfusion therapy, which includes endovascular methods (angioplasty with or without stent implantation) and vascular surgery methods (such as vascular bypass grafting).

Brahmbhatt et al. analyzed data from 24,645 patients undergoing reperfusion treatment in the lower limbs, 92% of patients with the open method and 8% with the endovascular method. It was found that female gender and Frailty were significant risk factors for complications of reperfusion therapy. Frailty was defined as mFI greater than 0.25 [78].

Gonzalez et al. performed the retrospective analysis of data from 431 patients suffering from PAD, treated by open vascular surgery (188 individuals, 43.6%) or endovascular intervention (243 individuals, 56.4%). For both groups analyzed together, it has been confirmed that Frailty severity measured by the mFI independently predicted major amputation (HR 2.16, 95% CI: 1.06–4.39), mortality (HR 2.62, 95% CI: 1.17–5.88), and the composite outcome of amputation and/or death (HR 1.97, 95% CI: 1.06–3.68). When analyzed separately, Frailty severity was found to be an independent predictor of limb loss only in the group treated by endovascular intervention (OR 6.28, 95% CI: 1.42–27.72). In this study, also such biomarkers of Frailty as absolute neutrophil count, blood albumin concentration, estimated glomerular filtration rate, hemoglobin concentration, serum creatinine concentration, age at time of procedure, body mass index (BMI), and polypharmacy have been assessed. Higher hemoglobin and albumin concentration were associated with significantly lower risk of amputation [28].

Rothenberg et al. presented the results of a study assessing the effect of Frailty on mortality of various interventional procedures in the field of vascular surgery, including suprainguinal and infrainguinal revascularization. The severity of patient’s Frailty was assessed using the Risk Assessment Index (RAI), the calculation of which takes into account data such as age, sex, disseminated cancer, weight loss, renal failure, congestive heart failure, dyspnea, functional status, cognitive impairment, and living status. Of the 139,569 patients enrolled in the study, 42,078 underwent infrainguinal revascularization (82.9% by the open method and 17.1% by the endovascular method) and 15,535 were subjected to suprainguinal revascularization (83.4% by the open method and 16.6% by the endovascular method). For suprainguinal revascularizations, 30-day mortality was 13.9% for very frail patients, 10.0% for frail patients and 1.8% for non-frail patients. For infrainguinal revascularizations, 30-day mortality was 9.4% for very frail patients, 6.3% for frail patients and 1.1% for non-frail patients [79].

Wojtasik-Bakalarz et al. presented the results of an interesting study in which they had assessed the influence of diabetes mellitus and coronary artery disease on the long-term follow-up in patients with PAD treated by retrograde recanalization of the femoropopliteal arterial region. Eighty-six patients with superficial femoral artery chronic total occlusion and after at least one unsuccessful attempt of antegrade recanalization have been enrolled. Although it has been proven that there is no significant difference in occurrence of major adverse peripheral events between people with diabetes and without diabetes, in the group with diabetes all-cause mortality was significantly higher. Major adverse peripheral events were defined as target vessel reintervention, nontarget vessel reintervention, and amputation [80].

Thus, there are data showing that the presence of Frailty may be associated with a worse prognosis and reduced effectiveness of reperfusion therapy, but further research is needed.

## 6. Lower Limb Amputation

Lower limb amputation is one of the most serious complications of PAD and its prevention is one of the most important goals of treatment.

Fang et al. documented that Frailty assessed by the Modified Frailty Index (mFI) in patients who had undergone lower extremity amputation is associated with significantly increased risk of readmission in 30-day period after operation but there is no difference in 30-day mortality in frail and non-frail population [81]. Campbell et al. in a paper published in 2001 have shown that “general Frailty” is associated with increased mortality in amputees but it is unclear what criteria for this term have been used [82].

It has been well documented that patients who have undergone lower limb amputation due to peripheral arterial disease suffer from more social and emotional problems than non-amputees [83]. Thompson et al. showed that quality of life patients treated by femorodistal bypass is significantly better than among patients who had undergone amputation [84]. Depression, perceived prosthetic mobility, social support, comorbidity, prosthesis problems, age and social activity participation have been elucidated as predictors of quality of life among individuals who had undergone lower limb amputation and had used a prosthesis for at least six months [85]. In another study, employment status, assistive devices, phantom-limb pain and residual stump pain were identified as factors affecting quality of life of amputees [86]. There is no significant difference in change of quality of life between patients with diabetes who underwent partial foot or transtibial amputation [87].

Helm et al. presented results showing that amputation can significantly affect the patient’s dependence and deteriorate functional capacity, which is a significant contribution to the development of the frailty syndrome. The emergence of these phenomena and their degree are also influenced by age, chronic diseases and the state before surgery [88].

The problem of lower limb amputation due to complications of chronic ischemia and its prevention is therefore an extremely important issue. Accurate characterization of modifiable risk factors for amputation due to complications of peripheral arterial disease also in patients with diabetes plays therefore the crucial role. Interesting data linking the issue of pharmacotherapy and the degree of diabetes control with the risk of amputation are available in the literature, and we focus on these issues later in this section.

The results of the CANVAS trial showed that although canagliflozin significantly reduces the risk of cardiovascular events, it unexpectedly increases the risk of lower limb amputation compared with placebo (HR 1.97, 95% CI: 1.41–2.75) [89]. Association between SGLT2 inhibitors exposure and increased risk of amputation was not confirmed in a meta-analysis and systematic review published in 2020 [90]. Notably, another meta-analysis published in 2020 that took into consideration the results of five randomized controlled trials documented that canagliflozin, as well as empagliflozin and dapagliflozin, did not increase the risk of lower limb amputation [91]. The results of a large multicenter observational study including SGLT2 inhibitors users (207,817) and DPP-4 inhibitors users (207,817) showed no significant difference between risk of below knee lower limb amputation between persons using SGLT2 inhibitors and DPP-4 inhibitors (HR 0.88, 95% CI 0.71–1.09). The mean exposed follow-up time for the matched cohort was 11 ± 9 months [92]. Dapagliflozin use is associated with similar risk of amputation compared with placebo [93].

Metformin use in patients with type 2 diabetes mellitus is associated with a lower below-the-knee arterial calcification score [94]. Metformin also improves some markers of endothelial function (vWF and sVCAM-1), but not all [95]. Studies in a mouse model show that, through a mechanism related to the AMPK/eNOS pathway, metformin may support revascularization treatment of critical lower limb ischemia [96]. It has been shown that although metformin reduces risk of adverse cardiac events and improves survival in patients with PAD, it did not have an impact on limb salvage rates after surgical or endovascular revascularization [97].

The FIELD study has documented that fenofibrate may decrease risk of amputation in patients with type 2 diabetes mellitus. A total of 9795 individuals participated in this randomized placebo-controlled trial. The time of follow-up was 5 years. The risks of first amputation (45 vs. 70 events; HR 0.64, 95% CI: 0.44–0.94, *p* = 0.02) and minor amputation events without known large-vessel disease (18 vs. 34 events; HR 0.53, 95% CI: 0.30–0.94; *p* = 0.027) were lower for patients assigned to fenofibrate than for patients assigned to placebo, with no difference between groups in risk of major amputations (24 vs. 26 events; HR 0.93, 95% CI: 0.53–1.62; *p* = 0.79) [98]. Li et al. conducted an interesting study on mice to explain the mechanism of the phenomenon observed in the FIELD study. They have used male adiponectin-deficient mice and their wild-type littermates. It was concluded that fenofibrate could promote revascularization in response to ischemia through adiponectin-dependent AMPK signaling. It has been shown that fenofibrate stimulates the phosphorylation of AMPK and eNOS in the ischemic muscles in wild type mice but not in adiponectin knock-out mice [99]. It had been described previously that adiponectin stimulated angiogenesis in response to tissue ischemia [100].

In patients with critical limb ischemia who underwent revascularization treatment statin therapy is associated with increased rate of limb salvage compared with patients not treated with a statin in 24-months period (83% vs. 62%, *p* = 0.001) [101]. Morimoto et al. suggested that the Akt/MDM2/p53 pathway may play an important role in limb salvage associated with atorvastatin therapy in type 2 diabetes [102]. There is no significant difference in amputation-free survival between patients with PAD who were referred for peripheral angiography or endovascular procedure treated by high-intensity statin dose versus low- or moderate-statin dose but high-intensity statin therapy is associated with improved survival and fewer major adverse cardiovascular events compared with low- or moderate-statin therapy [103].

McGinigle et al. proved that within 30 days after bypass implantation due to symptomatic peripheral arterial disease in patients with diabetes mellitus, suboptimal blood glucose levels measured by the percentage of glycated hemoglobin is associated with an increased risk of complications, including amputation [104]. According to Singh et al., percentages of glycated hemoglobin exceeding 8% in patients who underwent infrainguinal lower extremity bypass surgery are associated with significantly increased risk of major limb adverse events (such as amputation above the ankle and loss of primary graft patency) only in the subgroup without critical limb ischemia [105].

Avoiding lower limb amputation is one of the most important goals in the treatment of chronic lower limb ischemia. Research shows that some drugs used to treat diabetes and lipid disorders as well as control of blood glucose concentration can modify this risk. This is an interesting area for further research, both at the clinical and pre-clinical level.

## 7. Frailty Assessment in Clinical Practice

For the purposes of routine clinical practice, it is important to develop optimal methods of assessing the severity of Frailty in patients, which would allow for the most accurate prediction of its impact on the prognosis and effectiveness of treatment, and this could facilitate diagnostic and therapeutic decisions in patient care.

Cachexia/Frailty has been shown to be the most powerful predictor of all-cause mortality in the group of 101 patients undergoing nonemergent unprotected left main percutaneous coronary intervention (unadjusted HR 14.0, 95% CI: 5.4–36.0) [106]. In a study performed in the Mayo Clinic in which 629 elderly people were enrolled, it was proved that Frailty, according to Fried’s criteria, was associated with significantly increased 3-year mortality in comparison to non-frail individuals (28% vs. 6%) [107].

It has been shown that home-based preoperative rehabilitation (PREHAB) may improve functional ability and reduce hospital length of stay for frail patients undergoing cardiac surgery (CABG or valve surgery) [108]. This suggests that the assessment of the severity of Frailty and the use of perioperative management adequate for this group of patients may have a positive effect on the prognosis.

It should be noted that the currently available Frailty assessment tools are not ideal. For example, among the commonly used criteria according to Fried there is walking speed, which reduces their usefulness for people who cannot move independently due to disabilities. Drey et al. postulated that before determination of Frailty according to Fried’s criteria, the patient should be examined for depressive disorders that may distort the assessment of the exhaustion criterion [109]. Fried defined slowness as velocity in the walking test not exceeding 0.65 m/s or 0.76 m/s (depending on body height) measured at a distance of 15 ft (4.57 m) [13]. It has been proposed that, for Frailty assessment, the velocity for safely crossing a signaled zebra crossing not exceeding 1.22 m/s in the United States [110] and 1.07 m/s in the United Kingdom [111] be used. Purser et al. presented the results of the study, showing that the assessment of the prevalence of Frailty in patients with multivessel coronary heart disease varies widely, depending on the criteria used. In a group of 309 study participants, the prevalence of Frailty was assessed as 27% according to the Fried scale, 50% with gait speed <0.65 m/s, and 63% according to the Rockwood scale [112]. This shows that the assessment of Frailty according to the different criteria available varies considerably. Taken together, it is a high priority to define optimal tool to assess Frailty in clinical practice and to determine cutoff value [113]. Poh and Teo presented an interesting analysis of the available tools for assessing the severity of Frailty in the elderly undergoing surgical treatment and proposed an algorithm for their use, which, however, must still be validated [114].

In routine clinical practice, specific diagnostic and therapeutic decisions are most often made according to guidelines and recommendations of scientific societies, formulated on the basis of the results of scientific research (the most valuable of which are the results of meta-analyses and randomized clinical trials) in accordance with the principles of Evidence-Based Medicine. It is worth noting that the problem of Frailty has been mentioned in the current guidelines of the European Society for Vascular Medicine. It was noted that the general principles and the recommendations described are also applicable to the elderly, including those with developed Frailty. The authors emphasized, however, that in people from this population, the expected benefit-risk ratio in relation to planned intervention treatment should be carefully assessed, taking into account the prolonged recovery period in patients with Frailty [11]. The development of detailed algorithms using the assessment of the severity of Frailty in the decision-making process on the qualification of the patient for invasive treatment of cardiovascular diseases may be an interesting direction of future research.

An important issue is also the development of standards of treatment in frail patients living with diabetes. It is worth noting that polypharmacy should be avoided, which also contributes to the development of frailty syndrome, but can also be a consequence of it [115]. The mentioned treatment standards should be understood as not only pharmacological treatment, but most of all an optimal set of physical exercises [33].

Therefore, it seems that the main problem in using the knowledge about frailty syndrome in everyday clinical practice is the lack of unambiguous, commonly accepted diagnosis criteria. The development of such criteria and their clinical validation in the context of the prognosis and effectiveness of the treatment of peripheral arterial disease using randomized clinical trials and meta-analyzes would allow the inclusion of the patient’s assessment of Frailty in the guidelines of medical practice developed in accordance with the principles of Evidenced Based Medicine.

## 8. Conclusions

The most important conclusions from this review of the literature on the association of peripheral arterial disease with Frailty in patients living with diabetes are presented in Table 4. The frailty syndrome is a phenomenon of increasing clinical importance, especially in developed countries with an aging population. This issue is of increasing interest to researchers. The results of the studies cited in our paper show that the characteristics of Frailty may affect the clinical course, treatment effectiveness and complications of chronic lower limb ischemia and on the other hand symptoms and complications of peripheral arterial disease may be associated with greater susceptibility to the development of Frailty. It should be noted that there is a need to further develop and improve the ability to assess the severity of Frailty, including in patients undergoing invasive treatment with interventional angiology or vascular surgery due to chronic lower limb ischemia.

The overall clinical picture, including the assessment of the severity of Frailty, and the estimation of the risk-to-benefit ratio, should be taken into account when selecting patients for treatment, taking into consideration rehabilitation, pharmacotherapy and interventional procedures. The most basic way in the treatment of PAD is physical activity (walking training). Severity of Frailty should be taken into account during preparation of exercise plan. From the point of view of everyday clinical practice, it seems reasonable that patients with clearly severe features of Frailty who do not have critical lower limb ischemia will more often be qualified for non-invasive treatment, because for them even a relatively short distance of claudication will be satisfactory for the needs of everyday situations, while in the case of people who, even in their old age, do not have severe Frailty, with claudication distance that limits their daily activity, will be better candidates for reperfusion treatment. Diabetes mellitus is a very strong risk factor for the development of cardiovascular diseases, so in the population of patients living with diabetes it may be particularly important.

## Figures and Tables

**Table 1:** Classification of claudication severity according to Fontaine and Rutherford scales [42,43,44].

Stage According to Fontaine Classification			Category According to Rutherford Classification
I	Asymptomatic		0
IIA	Claudication at distance > 200 m	Mild claudication	1
IIB	Claudication at distance < 200 m	Moderate claudication	2
		Severe claudication	3
III	Rest pain		4
IV	Necrosis	Minor tissue loss	5
		Major tissue loss	6

**Table 2:** Prevalence of Frailty depending on the ABI value [70].

ABI	Prevalence of Frailty
<0.9	17.5%
≥0.9 and <1.1	6.7%
≥1.1 and <1.4	4.7%
≥1.4	7.3%

**Table 3:** Distribution of ABI among no frail, pre-frail and frail participants of the Toledo Study for Healthy Aging [71].

ABI	No Frail	Pre-Frail	Frail
≤0.9	18.0%	19.0%	28.3%
0.9–1.0	25.2%	26.7%	28.3%
1.0–1.4	54.6%	52.1%	40.4%
>1.4	2.3%	2.1%	3.0%

**Table 4:** The most important findings of our review of the literature.

Frailty is an important clinical problem of modern medicine that is associated with a more severe course of some diseases and with an increased predisposition to the development of additional health problems. The pathogenesis of Frailty is multifactorial.
The 11-factor modified Frailty Index (mFI) is often used to assess Frailty in angiology and vascular surgery.
An ankle-brachial index lower than 0.9 is a criterion for the diagnosis of peripheral arterial disease, and also indicates a greater risk of overall cardiovascular disease. The results of epidemiological studies show that a reduced ABI value is also associated with a greater predisposition to Frailty.
Diabetes mellitus is associated with a greater predisposition to increased vascular stiffness; therefore, the diagnostic value of the ankle-brachial index is lower in this population and the toe-brachial index should also be assessed.
There is a need for further research to confirm the relationship between the toe-brachial index value and the predisposition to Frailty.
The severity of the frailty syndrome may influence the effects of reperfusion treatment of chronic lower limb ischemia, although the results so far are not conclusive in all aspects; therefore, further research is needed.
There is a need to develop uniform, precise criteria for the diagnosis and assessment of the severity of Frailty, adequate both for the needs of research and clinical practice.
The CANVAS study showed that the antidiabetic drug canagliflozin may increase the risk of amputation. However, later studies, including meta-analyses, did not confirm such a relationship.
Assessment of the occurrence and severity of Frailty should be included in the care of patients with diabetes and chronic ischemia of the lower limbs in the selection of adequate management, both in terms of walking training, pharmacological treatment and revascularization treatment. More research is needed in this area.

## Abbreviations

ABI	ankle-brachial index
Akt	protein kinase B
AMPK	5’-adenosine monophosphate-activated protein kinase
APN-KO	adiponectin knock-out
CABG	coronary artery bypass grafting
CI	confidence interval
CLI	critical limb ischemia
DPP-4	dipeptidyl peptidase-4
eNOS	endothelial nitric oxide synthase
Frailty	the frailty syndrome
HR	hazard ratio
IMT	intima-media thickness
MDM2	mouse double minute 2 homolog
mFI	modified Frailty Index
NET	neutrophil extracellular trap
NFAT	nuclear factor of activated T cell
NLRP3	NOD-, LRR- and pyrin domain-containing protein 3
OR	odds ratio
PAD	peripheral arterial disease
p53	tumor suppressor p53
RAI	Risk Assessment Index
SGLT2	sodium/glucose cotransporter 2
sVCAM-1	soluble form of vascular cell adhesion molecule 1
TBI	toe-brachial index
vWF	von Willebrand factor
WT	wild type
